# Commentary: Will genomics revolutionise research on gene–environment interplay?

**DOI:** 10.1111/jcpp.13687

**Published:** 2022-08-25

**Authors:** Robert Plomin, Essi Viding

**Affiliations:** ^1^ Institute of Psychiatry, Psychology and Neuroscience King's College London London UK; ^2^ Division of Psychology and Language Sciences University College London London UK

**Keywords:** gene‐environment correlation, gene‐environment interaction, polygenic scores, quantitative genomics

## Abstract

The synthesis of quantitative genetics and molecular genetics is transforming research in the behavioural sciences. The ability to measure inherited DNA differences directly has led to polygenic scores and to new methods to estimate heritability and genetic correlations. This issue provides examples of how these advances can be appllied to research on gene‐environment interplay in developmental psychopathology.

This special issue on ‘new horizons’ in the study of gene–environment interplay in developmental psychopathology is a real pot of gold at the end of a rainbow arcing back a century to the origins of the two worlds of genetics, quantitative genetics and molecular genetics. The two worlds began with a collision. When Mendel's laws were rediscovered in the early twentieth century, Mendelians, forerunners of molecular geneticists, looked for, and thought they found, the 3:1 segregation ratios indicative of single‐gene disorders according to Mendel's laws of heredity. The early quantitative geneticists thought that Mendel's laws of heredity were peculiar to the pea plant because they knew that most traits were continuously distributed.

The two worlds went their own way for most of the century because of their different goals, despite a 1918 paper showing how the two worlds of genetics are compatible (Fisher, 1918). In this foundational paper on quantitative genetics, Fisher showed how Mendel's laws of heredity could apply to complex traits if several genes, operating according to Mendel's laws, affected the traits. The century‐long arc is that the two worlds of genetics have finally come together after recognising that the heritability of complex traits is due to thousands of inherited DNA differences of miniscule effect sizes (Visscher, Yengo, Cox, & Wray, 2021).

## Quantitative genetics and molecular genetics

Some history is needed to appreciate the pot of gold at the end of this rainbow, which is evidenced by this special issue. For a century, the goal for molecular geneticists was to understand genes; for quantitative geneticists, the goal was to understand phenotypes. Molecular geneticists investigated mutations and dichotomous disorders, while quantitative geneticists studied naturally occurring variation and continuous dimensions.

The first quantitative genetic twin and adoption studies were conducted 100 years ago and slowly led to a mountain of data showing that all traits were significantly and substantially heritable (Polderman et al., 2015). The same research provided strong evidence for the importance of environmental influence because heritabilities are only 50 per cent on average. A major advance in the study of gene–environment interplay was the incorporation of measures of the environment in quantitative genetic designs, which led to two important discoveries. First, most measures of the environment widely used in psychology show genetic influence; heritabilities are about 25% on average (Kendler & Baker, 2007; Plomin & Bergeman, 1991). This indicates genetic involvement in environmental exposures. In other words, many environmental risk and protective factors, such as maternal negativity/positivity, are partly a by‐product of genetically influenced traits and behaviours (McAdams, Gregory, & Eley, 2013). Second, most associations between environmental measures and psychological traits are significantly mediated genetically, about 50% on average, again indicating gene–environment correlation (Plomin, 1994). In line with the findings from twin studies, adoption studies comparing associations between family environment and children's development in nonadoptive and adoptive families find evidence for passive gene–environment correlations, because these associations are stronger in nonadoptive (biological) than adoptive families. Further corroboration comes from studies using the children‐of‐twins design (Knopik, Neiderhiser, DeFries, & Plomin, 2017).

Since the 1920s, molecular genetics had made great progress in studying single‐gene disorders and mapping them to chromosomes. By the 1940s, it was clear that DNA is the mechanism for Mendel's laws of genetics and in 1953 the most famous paper in biology was published showing the double helix structure of DNA (Watson & Crick, 1953). In the 1960s, the DNA code was cracked showing that three‐letter sequences of the four‐letter alphabet of DNA coded for the 20 amino acids.

The dawn of the genomics revolution began in the 1970s with the ability to sequence DNA. This led to the Human Genome Project and the discovery of millions of variants in inherited DNA. Genotyping these DNA variants was facilitated by the development of polymerase chain reaction, which could create millions of DNA copies even from a single cell's DNA.

This development ushered in the 1990s decade of candidate gene research. Because it was expensive and time‐consuming to genotype DNA variants, researchers studied a few genes thought to be good candidates, usually neurotransmitter genes in the case of psychopathology. Thousands of such studies were published and hundreds used candidate genes to study gene–environment interaction. However, few of these candidate gene associations or gene–environment interactions replicated. Most notably, these studies were woefully underpowered to detect the tiny effect sizes that we now know to be responsible for heritability. Their lack of power was often further compounded by the poor quality of the environmental measures available in many samples.

## Quantitative genomics

We now know that heritability is due to many associations of small effect size, as Fisher predicted in 1918. We have been able to confirm this because of a technological advance. The tipping point for the genomics revolution came in the 2000s with the invention of the DNA microarray that could genotype hundreds of thousands of DNA variants (single‐nucleotide polymorphisms, SNPs) in a few hours, accurately and inexpensively. Instead of studying a few candidate genes hypothesised to affect a trait, this technological advance of the ‘SNP chip’ made it possible to take an empirical approach to gene‐finding by assessing associations throughout the genome, called genome‐wide association.

In 2007, the first major genome‐wide association study of seven common disorders, the Wellcome Trust Case Control Consortium, was successful in identifying replicable associations, with the unfortunate exception of the only behavioural disorder, bipolar disorder (The Wellcome Trust Case Control Consortium, 2007). It led to a tsunami of genome‐wide association studies that have identified hundreds of thousands of SNPs associated with complex physical, physiological and psychological traits.

A surprising outcome of genome‐wide association studies is that the biggest effects were much smaller than anyone expected, often accounting for just 0.0002 of the variance. This means that hundreds of thousands of DNA differences are responsible for the heritability of complex traits. Recognition of this extreme polygenicity was the tipping point that brought the two worlds of genetics together to create a synthesis that could be called quantitative genomics. This completes the century‐long arc from Fisher's foundational paper on quantitative genetics in 1918 (Visscher et al., 2021).

Quantitative genomics is the pot of gold for gene–environment interplay because it enables direct measurement of inherited DNA differences. All of 9 papers in this special issue involve quantitative genomics. In contrast, less than 10 years ago, none of the 11 papers in the 2013 *JCCP* special issue on gene–environment interplay involved quantitative genomics, although several of them employed candidate genes (Petrill, Bartlett, & Blair, 2013).

## Polygenic scores

Quantitative genomics has used measured DNA variants from genome‐wide association studies in two ways, as exemplified in this special issue. The first was to realise that the thousands of tiny effects of SNP associations from genome‐wide association studies can be aggregated, like items on a scale, to create composites called polygenic scores. Hundreds of polygenic scores are available for use in any sample of unrelated individuals from whom DNA and SNP chip genotyping has been obtained. Twins or adoptees are not needed to assess genetic influence indirectly; DNA differences can be measured directly with polygenic scores. Although huge sample sizes are needed for genome‐wide association studies to detect these tiny effects, polygenic scores, once generated from the results of genome‐wide association studies, can be used in studies with modest sample sizes.

Papers in this special issue investigate polygenic scores for schizophrenia and major depression (Machlitt‐Northen et al., 2022), suicide attempts (Lannoy et al., 2022), ADHD (Agnew‐Blais et al., 2022; Cheesman et al., 2022) and externalising problems (Kretschmer et al., 2022). These polygenic scores are used to investigate correlations and interactions with environmental measures as they affect behavioural traits. The environmental measures used in these studies include psychosocial risk factors, negative life events, schools, household chaos, family dysfunction and early life stress. The behavioural traits include suicidal ideation, educational achievement, ADHD symptoms, externalising behaviour and behaviour problems. They report several examples of gene–environment correlation (Agnew‐Blais et al., 2022; Kretschmer et al., 2022; Machlitt‐Northen et al., 2022).

It will take some time for the dust to settle on the explosion of research on gene–environment interplay using polygenic scores because of the thousands of possible comparisons between polygenic scores, environmental measures and behavioural traits. In the meantime, it will be important to avoid repeating the failures to replicate from the candidate gene era, such as looking at several combinations of polygenic scores, environmental measures and behavioural traits, and reporting only the significant findings. Replication is key.

Polygenic scores also enable novel analyses. A specific example in this issue finds that genetic effects differ across schools, an analysis not possible with twins or adoptees (Cheesman et al., 2022). A general example central to the study of gene–environment interplay involves the addition of parental polygenic scores, called trios when both parents and a child are included. The inclusion of parental polygenic scores makes it possible to control for parental polygenic scores to disentangle ‘direct’ and ‘indirect’ effects of polygenic scores on children's traits. Effects are called ‘direct’ to the extent that children's polygenic scores predict their traits independent of their parents' polygenic scores, thus controlling for passive gene–environment correlation in relation to the polygenic score. Indirect effects are those associated with the parents' polygenic scores on children's traits independent of the children's polygenic score. For example, Cheesman et al. (2022) controlled for ADHD polygenic scores of parents to investigate direct effects of children's ADHD polygenic scores.

Two papers in this issue show how parental polygenic scores can be extended to investigate gene–environment correlation incorporating measures of the environment. Agnew‐Blais et al. (2022) report that both mothers' and children's ADHD polygenic scores correlate with household chaos, suggesting gene–environment correlation. A novel analysis showed that children's ADHD polygenic score correlates independently with household chaos after controlling for mothers' ADHD polygenic score, suggesting that children contribute to household chaos by evocative or active gene–environment correlation. Machlitt‐Northen et al. (2022) report several examples of gene–environment correlation for polygenic scores for schizophrenia and major depressive disorder as well as evidence of a contribution of passive gene–environment correlation as indicated by indirect effects of parental polygenic scores. Kretschmer et al. (2022) also report some evidence for indirect effects suggesting evocative gene–environment correlation.

## Quantitative genomic estimates of heritability and genetic correlations

The second quantitative genomic advance was to use the hundreds of thousands of SNP genotypes for each individual to estimate heritability and genetic correlations directly from DNA rather than indirectly from twin and adoption studies. One method, called GCTA or GREML, relates random SNP differences across the SNP chip between pairs of unrelated individuals to each pair's trait differences, creating millions of pair‐by‐pair comparisons from samples of thousands of unrelated individuals (Yang et al., 2011). This method can estimate what is called SNP heritability, so called because it is limited to heritability as detected by the common SNPs genotyped on current SNP chips (as opposed to rare variants that can also contribute to heritability estimates, but require a different method of whole‐genome sequencing to detect) (Wainschtein et al., 2022). For example, Choi et al. (2022) in this issue found that SNP heritability is 19% for externalising problems and 6% for internalising problems. These estimates fall far short of the twin study estimates of heritability, which is one type of ‘missing heritability’. The other type of missing heritability is the even lower variance explained by polygenic scores, often less than 10% for behaviour problems. SNP heritability is the ceiling for genome‐wide association studies and for polygenic scores derived from them.

GCTA can also be used to estimate genetic correlations between traits. Choi et al. (2022) in this issue show how GCTA can be extended to estimate the effect of SNPs, multiple measured environments, and their interactions on behaviour problems. Another paper in this issue (Eilertsen et al., 2022) integrates GCTA with trios, called trio‐GCTA, and reports some indirect genetic effects of parents on children's ADHD symptoms and conduct problems, in addition to more substantial direct genetic effects.

A bonus of this special issue is the inclusion of two methodological papers. Allegrini et al. (2022) provide a guide to computing and implementing polygenic scores, with a focus on longitudinal applications. They also describe multi‐trait approaches to genome‐wide association and aggregated polygenic scores that increase predictive power, an important advance not represented in this special issue. However, these approaches will hopefully feature in future developmental studies, including those that make their way to the *JCPP*. Pingault et al. (2022) discuss the many complexities in causal modelling of gene–environment interplay.

Quantitative genomics has been a bonanza for methodologists, but we hope the complexities of causal modelling will not deter developmentalists from taking advantage of the DNA revolution. Until the invention of polymerase chain reaction and SNP chips, the major obstacle was the expense of collection and extraction of DNA and the process of genotyping many DNA variants for large samples. That process is now routinised and available inexpensively in many molecular genetic laboratories in universities as well as commercially. As discussed by Allegrini et al. (2022) the hurdle now is the construction of polygenic scores given the explosion of new techniques. However, we have no doubt that this process will also become routinised, as we are already beginning to see in the resources and pipelines mentioned by Allegrini et al. (2022).

However, the complexities of causal modelling described by Pingault et al. (2022) will remain. Trying to extract causality from data that are fundamentally correlational, especially given the complexities of the developmental interplay between genes and environment, is daunting, even with measured genotypes, measured environments and longitudinal designs. Instead, we suggest that many of the applications of polygenic scores will focus on prediction rather than explanation, such as intervening to prevent problems rather than just treating problems once they occur, personalised rather than one‐size‐fits‐all interventions, and focusing on dimensions rather than disorders (Plomin, 2018). A case can be made that polygenic scores can be useful in terms of prediction without regard to explanation (Plomin & von Stumm, 2022). Furthermore, accumulating evidence documenting gene–environment correlation has implications for intervention contexts, regardless of whether genotyping takes place or not. For example, interventions for conduct problems typically involve parent training, but if parents share some of the genetically influenced vulnerabilities of their child, this augments the challenge of delivering a systemic intervention. Responding to a child with dysregulated emotions and challenging behaviour is a lot more difficult if the parent also has difficulties in regulating their emotions. This means that the most vulnerable families will need substantial ‘scaffolding’ to support them and may require interventions at multiple developmental points.

The papers in this issue analysed data from large longitudinal cohort studies such as the Adolescent Brain and Cognitive Development (ABCD) Study (Choi et al., 2022), ALSPAC (Lannoy et al., 2022), the E‐risk study (Agnew‐Blais et al., 2022), Millennium Cohort Study (MCS) and the 1958 National Child Development Study (NCDS) (Machlitt‐Northen et al., 2022), the Tracking Adolescents' Individual Lives Survey (TRAILS) (Kretschmer et al., 2022), and the Norwegian Mother, Father and Child Cohort Study (MoBa) (Cheesman et al., 2022; Eilertsen et al., 2022). Most cohort studies have arrangements in place for making data available to researchers. Much quantitative genomic research will continue to be done collaboratively using data from such large studies. However, researchers with reasonably large and valuable samples would do well to consider getting DNA from cheek swabs, extracting the DNA and genotyping the DNA on SNP chips, which costs about £60 per individual. This can be done by most molecular genetic laboratories, as well as by companies such as 23andMe, at about the double the price, who can make genotyping results available to researchers with consent of the participants. The current problem, as discussed by Allegrini et al. (2022) in this issue, is that creation of polygenic scores is complicated so that collaboration with experts is advised until the pipelines become more routinised.

Instead of researchers doing this on their own, the trend is towards using centralised facilities such as the 13 NIHR Bioresource Centres in the United Kingdom. For desirable samples, they will cover the costs of getting, extracting, genotyping and storing DNA (https://bioresource.nihr.ac.uk/about‐us/). However, researchers need to relinquish considerable control of their samples by assuring open access to the data as well as obtaining consent from participants for re‐contact, use of medical records and commercial use of the data. Another development to watch is Our Future Health, which plans to do whole‐genome sequencing of 5 million individuals (https://ourfuturehealth.org.uk/about‐us/). Advantages of centralised processing include quality control and long‐term storage of DNA and data.

## Conclusion

The synthesis of quantitative genetics and molecular genetics is transforming research in the behavioural sciences. The ability to measure inherited DNA differences directly has led to polygenic scores and to new methods to estimate heritability and genetic correlations. This issue provides examples of how these advances can be applied to research on gene–environment interplay in developmental psychopathology. We are particularly excited to see multiple studies focusing on gene–environment correlation using measured genotypes and environments. This line of research has received less focus to date than gene–environment interaction studies, yet has substantial potential to help us understand developmental dynamics and contexts critical for mental health and wellbeing. As this research progresses, we would also urge researchers conducting gene–environment interaction studies to routinely examine that their findings are not partly due to gene–environment correlation.

There is of course no pot of gold at the end of a rainbow guarded by leprechauns. However, the amazing advances that have exploded from the fusion of the two worlds of genetics during the past decade seem almost as magical.
